# A Somatic Mutation Signature Predicts the Best Overall Response to Anti-programmed Cell Death Protein-1 Treatment in Epidermal Growth Factor Receptor/Anaplastic Lymphoma Kinase-Negative Non-squamous Non-small Cell Lung Cancer

**DOI:** 10.3389/fmed.2022.808378

**Published:** 2022-05-03

**Authors:** Jie Peng, Lushan Xiao, Dan Zou, Lijie Han

**Affiliations:** ^1^Department of Medical Oncology, The Second Affiliated Hospital, Guizhou Medical University, Kaili City, China; ^2^Hepatology Unit, Department of Infectious Diseases, Nanfang Hospital, Southern Medical University, Guangzhou, China; ^3^Department of Hematology, The First Affiliated Hospital of Zhengzhou University, Zhengzhou, China

**Keywords:** non-small cell lung cancer, anti-PD-1, best overall response, somatic mutations, EGFR/ALK negative

## Abstract

**Background:**

We aimed to exploit a somatic mutation signature (SMS) to predict the best overall response to anti-programmed cell death protein-1 (PD-1) therapy in non-small cell lung cancer (NSCLC).

**Methods:**

Tumor samples of 248 patients with epidermal growth factor receptor (EGFR)/anaplastic lymphoma kinase (ALK)-negative non-squamous NSCLC treated with anti-PD-1 were molecularly tested by targeted next-generation sequencing or whole exome sequencing. On the basis of machine learning, we developed and validated a predictive model named SMS using the training (*n* = 83) and validation (*n* = 165) cohorts.

**Results:**

The SMS model comprising a panel of 15 genes (*TP53, PTPRD, SMARCA4, FAT1, MGA, NOTCH1, NTRK3, INPP4B, KMT2A, PAK1, ATRX, BCOR, KDM5C, DDR2*, and *ARID1B*) was built to predict best overall response in the training cohort. The areas under the curves of the training and validation cohorts were higher than those of tumor mutational burden and PD-L1 expression. Patients with SMS-high in the training and validation cohorts had poorer progression-free survival [hazard ratio (HR) = 6.01, *P* < 0.001; *HR* = 3.89, *P* < 0.001] and overall survival (*HR* = 7.60, *P* < 0.001; *HR* = 2.82, *P* < 0.001) than patients with SMS-low. SMS was an independent factor in multivariate analyses of progression-free survival and overall survival (*HR* = 4.32, *P* < 0.001; *HR* = 3.07, *P* < 0.001, respectively).

**Conclusion:**

This study revealed the predictive value of SMS for immunotherapy best overall response and prognosis in EGFR/ALK-negative non-squamous NSCLC as a potential biomarker in anti-PD-1 therapy.

## Introduction

Although immune checkpoint inhibitors (ICIs) have shown considerable success in patients with non-small cell lung cancer (NSCLC) in recent years, unsatisfactory response rates are still a limitation (1, 2). In previous studies, several predictive biomarkers for successful immunotherapy including tumor mutational burden (TMB) (3, 4), gene expression profile (5), PD-L1 expression (6), tumor-infiltrating lymphocytes (7), and high microsatellite instability (8) have been reported. However, there were some prevalent limitations to these biomarkers. First, the different cut-off values for TMB or PD-L1 expression remain controversial. Second, tumor heterogeneity and unsatisfactory predictive accuracy have restricted the real-world clinical practice of current signatures, suggesting value in finding more useful and precise biomarkers.

An increasing number of studies have revealed that specific somatic variants are significantly associated with the immunotherapy response or resistance. For example, epidermal growth factor receptor (EGFR) mutation or *MDM2* amplification have been reported to be related to hyper-progressive disease (9). Furthermore, *STK11* and *B2M* are negatively associated with programmed cell death protein-1 (PD-1)/PD-L1 inhibitor resistance, resulting in poor responsiveness (10). Additionally, *TP53*, *KRAS*, and *POLE* mutations can predict the PD-1/PD-L1 blockade response in advanced NSCLC (11). Moreover, different commutations, including KL (*KRAS* and *STK11*) and KP (*KRAS* and *TP53*) molecular subtypes, showed diverse responses to ICIs in NSCLC (12, 13). Finally, multiple mutations in DDR or Notch1/2/3 pathways indicate favorable clinical prognosis and response in NSCLC patients receiving ICI therapy (14, 15). Thus, we speculated that a panel of somatic mutations could be exploited to build a robust predictive model for identifying patients who would acquire a favorable or poor response to immunotherapy in advanced NSCLC.

Considering the data mining of next-generation sequencing (NGS) and whole exome sequencing (WES) in EGFR/anaplastic lymphoma kinase (ALK) non-squamous NSCLC patients treated with ICIs, we used a routine machine learning model based on somatic mutation profiling to develop a genomic signature for predicting the best overall response (BOR) and the prognosis of immunotherapy. Such a classification pattern of genomic panels could serve as a useful and robust tool for selecting patients who would benefit from ICIs.

## Materials and Methods

### Immunotherapeutic Patients

Our databases were derived from three cohorts of patients with advanced NSCLC receiving ICI therapy. From the first cohort, 75 patients with stage IV NSCLC were treated with a combination of nivolumab and ipilimumab in the clinical trial CheckMate-012 (NCT01454102) between February 2013 and March 2015 (16). Sixteen patients with squamous cell lung cancer and 12 with EGFR/ALK-positive mutations were excluded. In total, 47 eligible patients were included in the current study from cohort 1. For the second cohort, there were 56 patients from the Dana-Farber Cancer Institute (DFCI) with metastatic NSCLC treated with anti-PD-1 treatment (8). Seven patients with squamous cell lung cancer and thirteen with EGFR/ALK-positive mutations were excluded. As a result, a total of 36 eligible patients from cohort 2 were included in the current study. From the third cohort, 240 patients receiving only anti-PD-1 or a combination of anti-CTLA-4 and anti-PD-1 treatments were retrospectively collected from the Memorial Sloan Kettering Cancer Center (MSKCC) between April 2011 and January 2017 (10). From this cohort, 38 patients with squamous cell lung cancer and 37 with EGFR/ALK-positive mutations were excluded. A total of 165 eligible patients were selected from the MSKCC. Finally, a total of 83 patients from the first and second cohorts were included in the training cohort, and the remaining 165 from the third cohort were included in the validation cohort. The institutional review board of the Second Affiliated Hospital of Guizhou Medical University approved our clinical research design. We have been conducted in accordance with the World Medical Association’s Declaration of Helsinki.

### Study Design

In this study, a three-step approach was used to develop and validate the somatic mutation signature (SMS) in advanced NSCLC patients undergoing immunotherapy. First, on the basis of the least absolute shrinkage and selection operator (LASSO) method, we used the somatic mutation profiles of the WES database to select the optimal gene panel for predicting BOR. Second, we used the pattern classification of the support vector machine (SVM) algorithm to build a predictive model of the SMS according to clinical treatment response and genomic mutation profiling in the training cohort. The somatic mutations could be computationally evaluated using the various mutational databases and the severity of the disease could also be predicted using the SVM algorithms. The SMS model was validated in the independent MSKCC cohort. All patients were divided into two groups (SMS-low and SMS-high) on the basis of the optimal cut-off of the receiver operating characteristic (ROC) and analyzed to predict progression-free survival (PFS) and overall survival (OS). Furthermore, the SMS model was analyzed in multivariate analyses of PFS and OS and the application of different clinical variables in the combination set.

### Best Overall Response, Progression-Free Survival, and Overall Survival

This research study aimed to examine the BOR, PFS, and OS. BOR was defined as a record of the best outcomes from the beginning of the study to the end of the treatment, which was confirmed after considering a variety of factors. Furthermore, it was evaluated using Response Evaluation Criteria in Solid Tumors (RECIST) version 1.1, including complete response (CR), partial response (PR), stable disease (SD), and progressive disease (PD). PFS was defined as the time from the start of ICI treatment to the first confirmation of PD with RECIST version 1.1 or death. OS is defined as the time from the start of ICI treatment until death or the last contact.

### Whole Exome Sequencing and Targeted Next-Generation Sequencing

Tumor and blood samples from WES and targeted NGS profiles were collected before immunotherapy. DNA was extracted from formalin-fixed paraffin-embedded tumor masses and blood samples from patients. The 83 samples from the CheckMate-012 study (*n* = 36) and DFCI (*n* = 47) were tested using WES profiling and the 165 from the MSKCC using targeted NGS profiling as follows.

WES: Illumina Rapid Capture Exome Target Bait Kit (38 MB), Agilent Sure-Select Human All Exon v2.0 (44 MB)/v4.0 (51 MB) created a complete library for capturing exons. To do so, the HiSeq 2000, 2500, or 4000 platform (Illumina, San Diego, California) was used to provide 150 times the average target coverage, extensive exon library, and paired reads (2 × 76 bp). For each sample, the Burrows-Wheeler Aligner was used to generate a normal BAM file, and the tumor sequence was compared to the human hg19 genome construct. The Genome Analysis Toolkit was used to analyze basic quality factor recalibration, indel recombination, and duplicate deletion. Indel calls were generated using Indelocator software.^1^ The TMB in each sample was defined as the total number of non-synonymous mutations, including SNVs and indels.

Targeted NGS (MSK-IMPACT): Targeted NGS analysis was performed as described in a previous study (10). After amplification and sequencing, the barcode library targeted exons and chose introns of 468 (13 patients, version 3), 410 (116 patients; version 2), or 341 (36 patients; version 1) genes. In all tumor samples, the average sequence index was 7,443, and the minimum coverage depth was 913. A custom pipeline was used to identify the somatic alterations in the tumor samples. To normalize somatic non-synonymous TMB on panels of different sizes, we divided the detected coding regions in each panel by the total number of mutations, which covered 0.98 megabases (Mb) of the 341, 1.06 Mb of the 410, and 1.22 Mb of the 468 gene panels.

### Tumor Mutational Burden and Programmed Cell Death-Ligand 1 Testing Analysis

On the basis of the results of WES and targeted NGS profiling, we defined a high TMB as > 10/Mb or total somatic non-synonymous as ≥ 200 and low TMB as ≤ 10/Mb or total somatic non-synonymous < 200. The 22C3 (DAKO), 28-8 (DAKO), and E1L3N (Cell Signaling, Danvers, MA) were performed according to the manufacturer’s instructions of three antibodies. We used the percentage of membranous staining in the tumor cells to evaluate the PD-L1 expression. In this study, high PD-L1 expression was defined as > 50% of tumor staining.

### Statistical Analyses

LASSO is used to select the optimal gene panel and build a predictive model by choosing the most important variables from high-dimensional features (17, 18). The purpose of LASSO is to construct a first-order penalty function to obtain a refined model *via* the final determination of some variable coefficient 0 for feature screening. In this study, a LASSO method based on fivefold cross-validation was used to select 15 non-zero coefficients. Then, the SMS based on an SVM algorithm was built to predict the immunotherapy response. The performance of the SMS model was then evaluated in the training and validation cohorts by using ROC analysis. The optimal cut-off value for predicting BOR was defined using the maximum Youden index.

A *X*^2^-test was used to compare the SMS score with the BOR in MSK cohorts. The “pROC” package was used to plot the ROC curves and evaluate the accuracy. A confidence interval (CI) of 95% for the area under the curve (AUC) was computed for the training and validation cohorts. The Kaplan–Meier curves of PFS and OS were analyzed and plotted using the “survminer” package. Additionally, multivariate Cox regression analysis was performed on five variables: SMS, age, sex, smoker status, PD-L1, and TMB using the “rms” package. The “Forestplot” software package was used to analyze and visualize hazard ratios (HRs) for PFS and OS in the SMS-low and SMS-high subgroups. All statistical analyses were performed using GraphPad Prism (version 7.01) and R software (version 3.5.1). Statistical significance was set at *P* < 0.05.

## Results

### Characteristics of Patients

**FIGURE 1:**
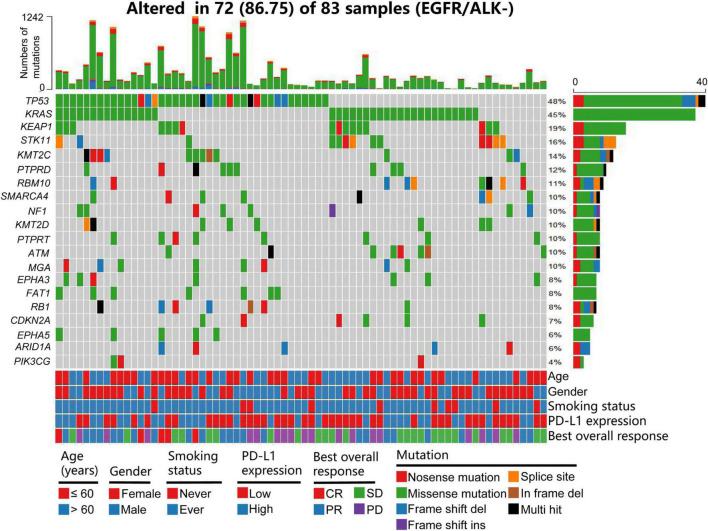
Summary of clinical and molecular features associated with response of ICI-based therapy in the training cohort with patients with EGFR/ALK-negative non-squamous NSCLC. Individual patients are represented in each column. Age is stratified as ≤ 60 years or > 6 years; sex as female and male; smoking status as ever and never; PD-L1 expression as 0–49% or ≥ 50%; and BOR as CR, PR, SD, and PD. Mutations include 7 mutational subtypes, and the TMB of each patient is calculated. The occurrences of top 20 genes in each case are represented in the OncoPrint. PD-L1, programmed cell death-ligand 1; NSCLC, non-small cell lung cancer; BOR, best overall response; CR, complete response; PR, partial response; SD, stable disease; PD, progressive disease; TMB, tumor mutation burden.

The basic characteristics of the patients in the training and validation cohorts are shown in Table 1. Of the patients, 47 (56.63%) in the training cohort and 106 (64.25%) in the validation cohort were more than 60 years old. A total of 48 (57.83%) patients in the training cohort and 89 (53.93%) in the validation cohort were female. Most patients in the training and validation cohorts (81.93 and 83.03%, respectively) had a history of smoking. Thirty-four (40.97%) patients in the training cohort and sixteen (9.7%) in the validation cohort had high PD-L1 expression (>50%). The samples of 104 (63.03%) patients were not tested for PD-L1 expression in the validation cohort. Patients with high TMB (>10/Mb or ≥ 200) accounted for 42.17% of the training and 34.55% of the validation cohorts. In the evaluation of BOR, 32 (38.55%) patients in the training and 39 (23.64%) in the validation cohorts achieved CR/PR. We found that the frequencies of PD-L1 expression and BOR were inconsistent between the training and validation cohorts (*P* < 0.001 and *P* = 0.014), but there were no significant differences in the other clinical characteristics between the two cohorts. Figure 1 shows a summary of the clinical and molecular features associated with the response of ICI-based therapy in the training cohort with EGFR/ALK-negative non-squamous NSCLC. Mutations include 7 mutational subtypes, and the occurrences of top 20 genes in each case are represented in OncoPrint.

**TABLE 1:** Characteristics of patients in the training and validation cohorts.

Variable	Training cohort (*n* = 83)	Validation cohort (*n* = 165)	*P*-value
Age (years)			0.230
≤ 60	36 (43.37%)	59 (35.75%)	
> 60	47 (56.63%)	106 (64.25%)	
Sex			0.560
Female	48 (57.83%)	89 (53.93%)	
Male	35 (42.17%)	76 (46.07%)	
Smoking status			0.828
Never	15 (18.07%)	28 (16.97%)	
Ever	68 (81.93%)	137 (83.03%)	
PD-L1 expression (%)			<0.001*
NA	0	104 (63.03%)	
≤ 50	49 (59.03%)	45 (27.27)	
> 50	34 (40.97%)	16 (9.7%)	
TMB			0.240
High	35 (42.17%)	57 (34.55%)	
Low	48 (57.83%)	108 (65.45%)	
Best overall response			0.014*
CR/PR	32 (38.55%)	39 (23.64%)	
SD/PD	51 (61.45%)	126 (76.36%)	

*P-value is derived from the difference between the training and validation cohorts in either of the clinical characteristics.*

*NA, not available; CR, complete response; PR, partial response; SD, stable disease; PD, progressive disease; TMB, tumor mutation burden.*

*P-value < 0.05.

### Development and Validation of Somatic Mutation Signature for Best Overall Response

**FIGURE 2:**
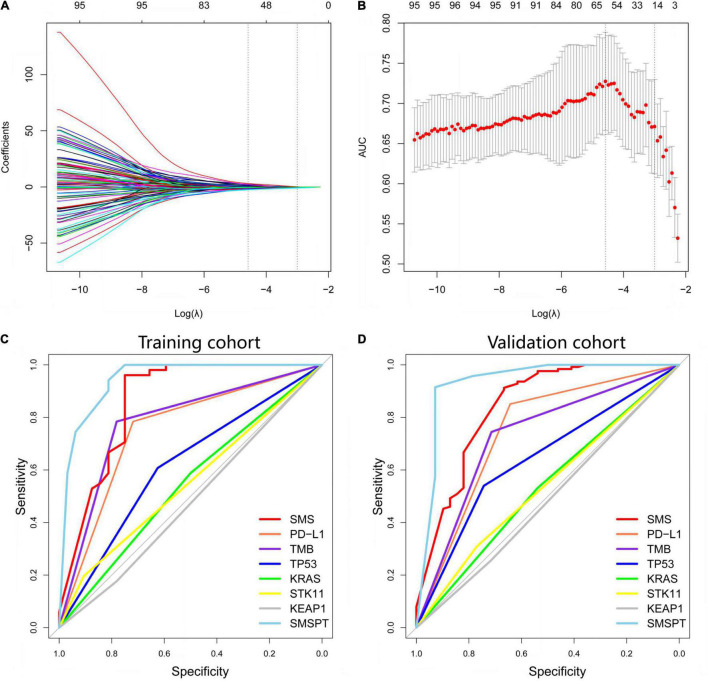
Selection of somatic mutation genes using the LASSO method and building the SMS model for BOR. **(A)** Fifteen non-zero coefficients are selected, and the coefficients are plotted against the log (λ) sequence. **(B)** LASSO coefficient analysis of the somatic mutation genes is shown by 10-fold cross-validation. The minimum value of log (λ) is −1.44 based on the 1-SE criteria. **(C,D)** Based on AUCs plotting, performances of SMS, PD-L1, TMB, *TP53, KRAS, STK11, KEAP1*, and SMSPT in the training and validation cohorts are presented. LASSO, least absolute shrinkage and selection operator; SMS, somatic mutation signature; BOR, best overall response; PD-L1, programmed cell death-ligand 1; TMB, tumor mutation burden; SMSPT, integrating SMS, PD-L1, and TMB; AUC, area under the curve.

On the basis of 5-fold cross-validation of LASSO, 15 genes with somatic mutations were selected and used to build a model of the SMS (Figures 2A,B). We used a gene panel of 15 somatic mutations to train the model by using the SVM method, and the SMS was built to predict the BOR after fine tuning. The SMS was compared with the *TP53/KRAS/KEAP1/STK11* driver genes, TMB, and PD-L1 expression in both cohorts (Figures 2C,D). We found that the SMS showed high AUC in the training and validation cohorts (AUC = 0.859, 95% CI: 0.767–0.951, sensitivity = 96.08%, 95% CI: 86.54–99.52%, specificity = 75.00%, 95% CI: 56.60–88.54%, *P* < 0.001; AUC = 0.841, 95% CI: 0.761–0.922, sensitivity = 91.27%, 95% CI: 84.92–95.56%, specificity = 61.67%, 95% CI: 49.78–80.91%, *P* < 0.001, respectively) (Supplementary Table 1). *TP53* mutation positively correlated with BOR in the training and validation cohorts (*P* = 0.075 and *P* < 0.001, respectively), and we did not find an association between *KRAS/KEAP1/STK11* mutations and BOR (*P* > 0.05 each). PD-L1 expression and TMB were also significantly associated with BOR in the training (AUC = 0.751, 95% CI: 0.639–0.863, sensitivity = 78.43%, 95% CI: 64.68–88.71%, specificity = 71.88%, 95% CI: 53.25–86.25%, *P* < 0.001; AUC = 0.817, 95% CI: 0.721–0.913, sensitivity = 78.43%, 95% CI: 64.68–88.71%, specificity = 78.13%, 95% CI: 60.03–90.72%, *P* < 0.001) and validation cohorts (AUC = 0.747, 95% CI: 0.585–0.908, sensitivity = 85.11%, 95% CI: 71.69–93.80%, specificity = 64.29%, 95% CI: 35.14–87.24%, *P* < 0.001; AUC = 0.657, 95% CI: 0.558–0.757, sensitivity = 67.46%, 95% CI: 58.54–75.54%, specificity = 64.10%, 95% CI: 47.18–78.8%, *P* = 0.002). Furthermore, we used a logistic model integrating the SMS, PD-L1, and TMB to build a combination model named SMSPT, which showed high AUCs in the training and validation cohorts (AUC = 0.937, 95% CI: 0.886–0.988, sensitivity = 94.12%, 95% CI: 83.76–98.77%, specificity = 81.25%, 95% CI: 63.56–92.79%, *P* < 0.001; AUC = 0.933, 95% CI: 0.833–1.000, sensitivity = 91.49%, 95% CI: 79.62–97.63%, specificity = 92.86%, 95% CI: 66.13–99.82%, *P* < 0.001, respectively) (Figures 2C,D and Supplementary Table 1).

### Somatic Mutation Signature Predicts Progression-Free Survival and Overall Survival in Patients With Immunotherapy

**FIGURE 3:**
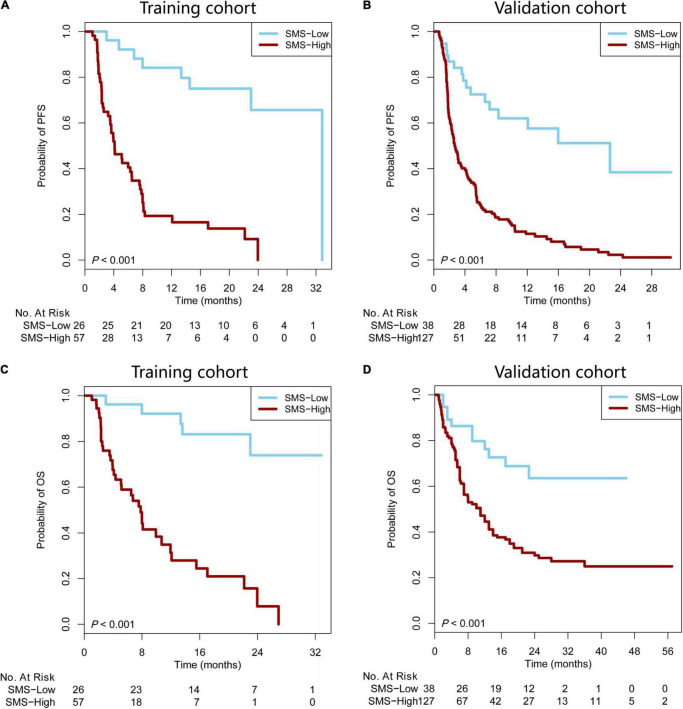
The SMS predicts PFS and OS in patients with immunotherapy. **(A,B)** Kaplan–Meier survival curves showing PFS between the SMS-low and -high groups in patients from the training and validation cohorts treated with anti-PD-1 therapy. **(C,D)** Kaplan–Meier survival curves showing OS between the SMS-low and -high groups in patients from the training and validation cohorts treated with anti-PD-1 therapy. SMS, somatic mutation signature; PFS, progression-free survival; OS, overall survival; NR, not reached; PD-1, programmed cell death 1.

According to the cut-off value (SMS scores = 1.95), our patients were stratified into SMS-high (>1.95) or SMS-low (≤ 1.95) groups. Compared with the SMS-low group in the training cohort with anti-PD-1 therapy, the SMS-high group showed a poorer median PFS (mPFS: 4.11 vs. 32.86 months) and OS [mOS: 7.81 months vs. not reached (NR)] [HR = 6.01 (3.54–10.20), *P* < 0.001; HR = 7.60 (4.12–14.03), *P* < 0.001, respectively] (Figures 3A,B). We then tested the SMS model in the validation cohort and found that the SMS-high group also presented a poorer median PFS (mPFS: 2.70 vs. 22.63 months) and OS (mOS: 11.00 months vs. NR) [HR = 3.89 (2.72–5.54), *P* < 0.001; HR = 2.82 (1.80–4.41), *P* < 0.001, respectively] than did the SMS-low group (Figures 3C,D). Considering 104 patients without PD-L1 expression tested in the validation cohort, we combined 83 from the training cohort and 61 from the validation cohort to build a combination cohort (*n* = 144) and performed a multivariate analysis of PFS and OS. We found that PD-L1, TMB, and the SMS were independent predictors of PFS in anti-PD-1 therapy (Table 2). Moreover, smoking status, PD-L1, TMB, and the SMS were also independent predictors of OS in this study.

**TABLE 2:** Multivariate analyses of PFS and OS in combination cohort (*n* = 144).

Variable	PFS		OS	
	HR (95% CI)	*P*-value	HR (95% CI)	*P*-value
Age (≤ 60 vs. > 60)	0.80 (0.52–1.21)	0.291	1.02 (0.68–1.54)	0.096
Sex (female vs. male)	0.84 (0.56–1.26)	0.417	0.70 (0.46–1.06)	0.910
Smoker status (never vs. ever)	1.24 (0.74–2.06)	0.397	1.958 (1.14–3.35)	0.014*
TMB (high vs. low)	0.44 (0.26–0.74)	0.002*	0.57 (0.35–0.92)	0.023*
PD-L1 (high vs. low)	0.28 (0.17–0.46)	<0.001*	0.36 (0.22–0.59)	<0.001*
SMS (high vs. low)	4.32 (2.32–8.06)	<0.001*	3.07 (1.71–5.49)	<0.001*

*PFS, progression free survival; OS, overall survival; HR, hazard ratio; CI, confidence interval; TMB, tumor mutation burden; SMS, somatic mutation signature.*

*P-value < 0.05.

### Applicability of Somatic Mutation Signature in Epidermal Growth Factor Receptor/Anaplastic Lymphoma Kinase-Negative Non-small Cell Lung Cancer Patients With Different Clinical Variables

**FIGURE 4:**
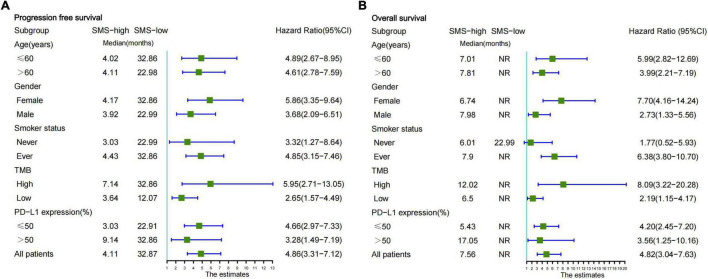
Subgroup analysis of the SMS for PFS and OS from the combination cohort according to basic clinical variables. **(A)** Forestplot of the SMS for PFS is presented in the combination cohort. **(B)** Forestplot of the SMS for OS is presented in the combination cohort. Each subgroup HR is computed from univariate analysis. Fixed-effects model is used to calculate pooled HRs for each subgroup. The bars indicate 95% CI. SMS, somatic mutation signature; PFS, progression-free survival; OS, overall survival; NR, not reached; PD-L1, programmed cell death-ligand 1; HR, hazard ratio; CI, confidence interval.

We further analyzed whether the SMS predictive model was feasible in specific groups of all EGFR/ALK-negative NSCLC patients. According to the basic clinical characteristics in the combination cohort, a univariate subgroup of PFS and OS was analyzed using the SMS (Figures 4A,B). The patients with SMS-high had significantly shorter PFS and OS regardless of age (≤ 60 vs. > 60 years) and sex (male vs. female). However, the SMS showed differentiated predictive values for anti-PD-L1 therapy in smokers. The SMS predicted the PFS and OS better in patients who are ever smoker than in patients who are never smokers. But the number of patients who never smoked was small (*n* = 27). Interestingly, we found that the SMS had good predictive ability in the subgroups of TMB and PD-L1 expression (Figures 4A,B). The SMS in the high TMB subgroup had better predictive ability for PFS and OS than that in the low TMB subgroup. In addition, the SMS had better predictive ability for PFS and OS in the low PD-L1 expression subgroup than in the high PD-L1 expression subgroup.

## Discussion

In this study, we found that the SVM classification of somatic mutations could predict BOR in patients with EGFR/ALK-negative NSCLC treated with anti-PD-1. In two independent cohorts, we found that the accuracy of the SVM model was greater than that of PD-L1 or TMB expression. In the two groups, patients treated with ICIs in the SMS-low group had better OS and PFS than those in the SMS-high group. We also found that the SMS model could predict prognosis in several clinical subgroups.

Previous studies have used sequencing techniques, including WES, WGS, and NGS, to analyze the association between genomic variants and prognosis (19–21). In most studies of immunotherapy (22, 23), specific genes were studied, and we found that it made it difficult to realize the precise predictive value for benefits from anti-PD-1 therapy in patients. In the current study, *TP53*, but not *KRAS*, mutations were positively associated with immunotherapy BOR in driver mutations, and *STK11* or *KEAP1* mutations were not significantly related to BOR. These results revealed that the single genomic mutation had weak predictive abilities for different molecular statuses, potentially resulting from tumor heterogeneity. Currently, WES and NGS testing of tumor tissue and blood samples have been used to quantify TMB in various solid tumors (24, 25). TMB is frequently calculated from the accumulation number of non-synonymous mutations, and high TMB has been reported to be correlated with a good response to anti-PD-1 therapy (3, 4, 26). However, the predictive value of TMB is controversial, and the accuracy is not satisfactory. Thus, we used the SMS model based on somatic mutations derived from targeted WES and NGS to determine BOR for anti-PD-1 therapy in patients with EGFR/ALK-negative NSCLC. The SMS classifications could precisely predict BOR and No-BOR in patients. We found that the SMS had a more accurate prediction than TMB and PD-L1 expression. Interestingly, the comprehensive model integrating the SMS, TMB, and PD-L1 expression had high predictive accuracy in both the training and validation cohorts. This indicates that not only TMB and PD-L1 expression testing but also fully mining mutation features based on machine learning is helpful in improving prediction ability. To help clinical practice, our model can be freely used online on a computer or mobile phone.^2^ Thus, the novel method is easy to use and could potentially screen patients with NSCLC for benefits from immunotherapy.

Previous studies show that through the use of immunotherapy for various cancers, patients who attained CR/PR frequently have better prognosis (27–29). We further analyzed the association between the SMS and prognosis and found that patients with SMS-low showed significantly longer PFS and OS than patients with SMS-high in both cohorts. This result suggests that the SMS model based on predicting BOR could effectively evaluate the clinical outcome of immunotherapy in the molecular subgroup of EGFR/ALK-negative NSCLC. In multivariate analyses of PFS and OS in the combination cohort, we found that the SMS, PD-L1 expression, and TMB were independent predictive factors, suggesting that the SMS model based on somatic mutations could be considered a novel biomarker for predicting prognosis. We also found that smoking status was an independent factor for OS. Patients who have smoked might have more mutant antigens causing lung cancer, which has been revealed in previous studies of immunotherapy (30, 31). Subgroup analysis of immunotherapy in patients with EGFR/ALK-negative NSCLC with SMS-low showed significantly better PFS than those with SMS-high. We found that the SMS model showed better prediction of OS in subgroups of smoking status (never), TMB-high, and PD-L1 low expression than in those of smoking status (ever), TMB-low, and PD-L1 high expression. Of note, the number of patients with smoking status “never” was relatively small. Additionally, all patients with SMS-low showed longer medium-OS time than those with SMS-high did. This indicated that our SMS model could serve as a well-stratified tool and improve the value of TMB or PD-L1 expression for predicting the prognosis in patients with EGFR/ALK-negative NSCLC receiving anti-PD-1/PD-L1 therapy.

Our study has three limitations. First, the sample size was relatively small, and the three cohorts were from American Medical Centers. Although the result of predicting response to immunotherapy was performed well, a large prospective study based on this SMS model should be tested across an international multicenter population in a clinical trial. Second, our study focused on mutational genes, and tumor heterogeneity might affect the results of genomic variants in WES or NGS. Thus, a multi-omics model, including tumor genomics, radiology, and pathology, should be considered to predict the response. Third, the sequencing tumor tissues were obtained from biopsy or surgery, and this was an invasive procedure. An SMS model based on the ctDNA of peripheral blood would be a non-invasive model that should be further investigated in the future.

Overall, our research supports the 15-gene SMS classification as a reliable prediction tool for identifying patients who may benefit from anti-PD-1 treatment in patients with EGFR/ALK-negative NSCLC. The new findings described in this study may help us develop a sequence database to explore new strategies for cancer immunotherapy. In the future, comprehensive pan-cancer research is needed to make better use of multigene SMS panels as predictive biomarkers for immunotherapy.

## Data Availability Statement

The datasets presented in this study can be found in online repositories. The names of the repository/repositories and accession number(s) can be found in the article/Supplementary Material.

## Ethics Statement

The studies involving human participants were reviewed and approved by the Second Affiliated Hospital of Guizhou Medical University. The patients/participants provided their written informed consent to participate in this study.

## Author Contributions

JP: conception, design, data analysis and interpretation, and administrative support. JP and LX: collection and assembly of data. JP, LX, DZ, and LH: manuscript writing and final approval of the manuscript. All authors contributed to the article and approved the submitted version.

## Conflict of Interest

The authors declare that the research was conducted in the absence of any commercial or financial relationships that could be construed as a potential conflict of interest.

## Publisher’s Note

All claims expressed in this article are solely those of the authors and do not necessarily represent those of their affiliated organizations, or those of the publisher, the editors and the reviewers. Any product that may be evaluated in this article, or claim that may be made by its manufacturer, is not guaranteed or endorsed by the publisher.

## Footnotes

1
http://archive.broadinstitute.org/

2
https://pengjie.shinyapps.io/Somatic/
