# Supplementary material for: A Somatic Mutation Signature Predicts the Best Overall Response to Anti-programmed Cell Death Protein-1 Treatment in Epidermal Growth Factor Receptor/Anaplastic Lymphoma Kinase-Negative Non-squamous Non-small Cell Lung Cancer
Source: Front Med (Lausanne). 2022 May 3;9:808378. doi: 10.3389/fmed.2022.808378 (PMC9112854; doi:10.3389/fmed.2022.808378)
Supplement: Supplementary file 1 [file Data_Sheet_1.docx]

**Supplementary materials**

# A Somatic Mutation Signature Predicts the Best Overall Response to Anti-PD-1 Treatment in EGFR/ALK-Negative Non-Squamous Non-Small Cell Lung Cancer

**Contents:**

**Table S1**

**Fig. S1**

| **Table S1. Performance of gene mutations, TMB, SMS and SMSPT in BOR prediction** | | | | |
| --- | --- | --- | --- | --- |
| **Groups** | **AUC**  **(95%CI)** | **Sensitivity (95%CI)** | **Specificity (95%CI)** | ***P* value** |
| **Training cohort** |  |  |  |  |
| *TP53 mutation* | 0.616 (0.491-0.741) | 60.78  (46.11-74.16) | 62.5  (43.69-78.9) | 0.075 |
| *KRAS mutation* | 0.544 (0.416-0.672) | 58.82  (44.17-72.42) | 50.00 (31.89-68.11) | 0.500 |
| *STK11 mutation* | 0.551 (0.425-0.676) | 19.61 (9.824-33.12) | 90.63 (74.98-98.02) | 0.434 |
| *KEAP1 mutation* | 0.521  (0.392-0.650) | 82.35 (69.13-91.6) | 21.88  (9.27-39.97) | 0.746 |
| TMB | 0.817  (0.721-0.913) | 78.43 (64.68-88.71) | 78.13 (60.03-90.72) | < 0.001* |
| PD-L1 expression | 0.751  (0.639-0.863) | 78.43  (64.68-88.71) | 71.88  (53.25-86.25) | < 0.001* |
| SMS | 0.859  (0.767-0.951) | 96.08  (86.54-99.52) | 75.00  (56.6-88.54) | < 0.001* |
| SMSPT | 0.943  (0.889-0.997) | 94.12  (83.76-98.77) | 81.25  (63.56-92.79) | < 0.001* |
| **Validation cohort** |  |  |  |  |
| *TP53 mutation* | 0.641 (0.545-0.738) | 53.97 (44.86-62.88) | 74.36  (57.87-86.96) | 0.007 |
| *KRAS mutation* | 0.535 (0.431-0.638) | 53.17 (44.08-62.12) | 53.87 (37.18-69.91) | 0.508 |
| *STK11 mutation* | 0.539  (0.437-0.641) | 30.95  (23.02-39.8) | 76.92 (60.67-88.87) | 0.458 |
| *KEAP1 mutation* | 0.514 (0.409-0.618) | 74.60  (66.08-81.93) | 28.21  (15.00-44.87) | 0.791 |
| TMB | 0.657 (0.558-0.757) | 67.46  (58.54-75.54) | 64.10  (47.18-78.8) | 0.002 |
| PD-L1 expression | 0.747  (0.585-0.908) | 85.11  (71.69-93.80) | 64.29  (35.14-87.24) | 0.005 |
| SMS | 0.841 (0.761-0.922) | 91.27 (84.92-95.56) | 61.67 (49.78-80.91) | < 0.001* |
| SMSPT | 0.933  (0.833-1.000) | 91.49  (79.62-97.63) | 92.86  (66.13-99.82) | < 0.001* |
| Abbreviations: AUC, area under the curve; CI, confidence interval; BOR, best overall response; SMS, somatic mutation signature; SMSPT, combination of SMS, PD-L1, and TMB; TMB, tumor mutation burden. **P* value < 0.05 | | | | |

**
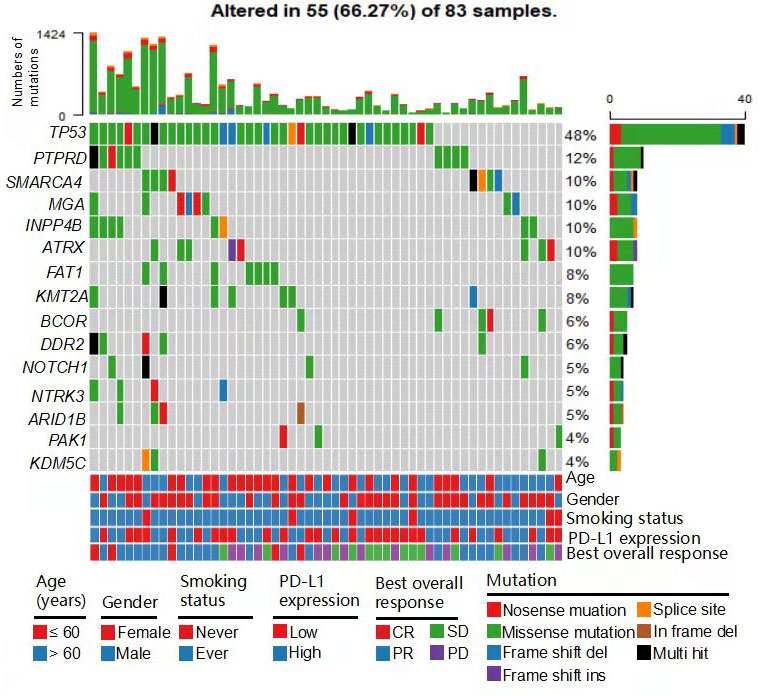
**

**Fig. S1 Summary of clinical and selecting 15 genes** **associated with response of ICI-based therapy in the training cohort with patients with EGFR/ALK-negative non-squamous NSCLC.**

Individual patients are represented in each column. Age is stratified as ≤ 60 years or > 6 years; sex as female and male; smoking status as ever and never; PD-L1 expression as 0%–49% or ≥50%; and BOR as CR, PR, SD, and PD. Mutations include 7 mutational subtypes, and the TMB of each patient is calculated. The occurrences of selecting 15 genes in each case are represented in the OncoPrint. Abbreviations: PD-L1, programmed cell death-ligand 1; NSCLC, non-small cell lung cancer; BOR, best overall response; CR, complete response; PR, partial response; SD, stable disease; PD, progressive disease; TMB, tumor mutation burden.
